# A New Berlin Questionnaire Simplified by Machine Learning Techniques in a Population of Italian Healthcare Workers to Highlight the Suspicion of Obstructive Sleep Apnea

**DOI:** 10.3389/fmed.2022.866822

**Published:** 2022-05-25

**Authors:** Giorgio De Nunzio, Luana Conte, Roberto Lupo, Elsa Vitale, Antonino Calabrò, Maurizio Ercolani, Maicol Carvello, Michele Arigliani, Domenico Maurizio Toraldo, Luigi De Benedetto

**Affiliations:** ^1^Laboratory of Biomedical Physics and Environment, Department of Mathematics and Physics “E. De Giorgi”, University of Salento, Lecce, Italy; ^2^Laboratory of Interdisciplinary Research Applied to Medicine, University of Salento, Local Health Authority, Lecce, Italy; ^3^“San Giuseppe da Copertino” Hospital, Local Health Authority, Lecce, Italy; ^4^Department of Mental Health, Local Health Authority, Bari, Italy; ^5^“Nuovo Ospedale degli Infermi” Hospital, Local Health Authority, Biella, Italy; ^6^Local Health Authority Marche Area Vasta 2 Health Department, Ancona, Italy; ^7^Brisighella Community Hospital, Local Health Authority, Romagna, Italy; ^8^Ear, Nose, and Throat Unit, “Vito Fazzi” Hospital, Local Health Authority, Lecce, Italy; ^9^Cardio-Respiratory Unit Care, Department of Rehabilitation, “Vito Fazzi” Hospital, Local Health Authority, Lecce, Italy; ^10^Integrated Therapies in Otolaryngology, Campus Bio-Medico University, Rome, Italy

**Keywords:** obstructive sleep apnea (OSA), Berlin questionnaire (BQ), risk factors, machine learning, simplified berlin questionnaire, screening test

## Abstract

Obstructive sleep apnea (OSA) syndrome is a condition characterized by the presence of repeated complete or partial collapse of the upper airways during sleep associated with episodes of intermittent hypoxia, leading to fragmentation of sleep, sympathetic nervous system activation, and oxidative stress. To date, one of the major aims of research is to find out a simplified non-invasive screening system for this still underdiagnosed disease. The Berlin questionnaire (BQ) is the most widely used questionnaire for OSA and is a beneficial screening tool devised to select subjects with a high likelihood of having OSA. We administered the original ten-question Berlin questionnaire, enriched with a set of questions purposely prepared by our team and completing the socio-demographic, clinical, and anamnestic picture, to a sample of Italian professional nurses in order to investigate the possible impact of OSA disease on healthcare systems. According to the Berlin questionnaire, respondents were categorized as high-risk and low-risk of having OSA. For both risk groups, baseline characteristics, work information, clinical factors, and symptoms were assessed. Anthropometric data, work information, health status, and symptoms were significantly different between OSA high-risk and low-risk groups. Through supervised feature selection and Machine Learning, we also reduced the original BQ to a very limited set of items which seem capable of reproducing the outcome of the full BQ: this reduced group of questions may be useful to determine the risk of sleep apnea in screening cases where questionnaire compilation time must be kept as short as possible.

## Introduction

Obstructive Sleep Apnea (OSA) is a syndrome characterized by partial or complete obstruction of the upper airways during sleep. This phenomenon, in turn, causes numerous and repetitive arousal from sleep to restore airways, leading to disrupted sleep, daytime hypersomnolence, and sympathetic activation. The obstruction of the airways may also lead to blood oxygen desaturation (1) during sleep, and cardiovascular lesions (2). OSA is associated with numerous conditions including stroke, hypertension and death (3, 4). These comorbidities are particularly evident in obese patients, and varying in severity according to gender and age.

The prevalence of OSA is highly different in the general population, ranging from 9 to 38%, with older age, male gender, and obesity as known risk factors (1, 5, 6). In advanced age groups, prevalence can even increase to 84% (1).

According to a worldwide epidemiological prevalence study (5) there are an estimated 936 million OSAS patients aged 30–69 years with mild-moderate OSA and 425 million patients aged 30–69 years with severe OSA who need Continuous Positive Airway Pressure (CPAP) treatment. In Italy, one study estimated the prevalence of moderate-to-severe OSA in 27% of the general population, with an overall prevalence of mild and moderate-to-severe OSA of more than 24 million people in the ages 15–74 years (54% adult population), while from a practical perspective, Italian NHS physicians diagnosed only 460,000 moderate-to-severe patients (4% of estimated prevalence) and 230,000 patients were treated (2% of estimated prevalence), highlighting a substantial gap between diagnosis and treatment. Considering that each patient is diagnosed many years after the onset of the disease, the direct and indirect healthcare costs determine a significant burden for the National Health System (NHS), which affects every single citizen. Prevention and early diagnosis are the only ways to achieve cost containment and improved quality of life.

Although studies have considerably increased in recent years, to date OSA is still a highly underdiagnosed disease. The gold standard for OSA diagnosis is nocturnal polysomnography (PSG) in the sleep laboratory. However, since this is not well workable for large numbers of patients, the Home Sleep Test (HST) is also an accepted validated ambulatory diagnostic method. Among non-invasive screening tools for OSA diagnosis in the general population, the Berlin questionnaire (BQ) (7) is the most widely used to define patients at risk for OSA. It was employed for the first time in the US: it contains ten questions related to risk factors and symptoms of OSA with the purpose of selecting high-risk OSA patients that may undergo polysomnography and increase the number of diagnosed patients.

The main purpose of this study was to find possible risk factors that are best correlated with being at high risk for OSA—according to the BQ—in professional nurses in order to investigate the possible impact of OSA on healthcare systems by considering one of the most important categories in health and assistance fields. We also assessed the capabilities of a reduced BQ of predicting a high-risk OSA group according to the result of the standard BQ. For this purpose, we used techniques related to supervised feature selection and Machine Learning.

## Methods

### Design

From May 2020 to September 2021 a cross sectional, multicenter study was conducted among professional nurses. Four hundred and five Italian subjects agreed to participate in the study. No eligible criteria were applied to the volunteers. The survey was conducted by means of an anonymous electronic questionnaire distributed on a voluntary basis. All subjects were asked to answer the BQ (7) and an additional set of 38 questions including items about baseline socio-demographic characteristics, work information, clinical status, and symptoms category. In particular, socio-demographic characteristics included gender, age, BMI, smoking, and neck circumference. For work information, we intended years of work experience, working hours, work shift, work shift regularity. For health status, we assessed the presence of arrhythmias, sleep disturbances, hypo/hyperthyroidism, anxiety, hypertension, transient ischemic attack or stroke, diabetes mellitus, chronic obstructive pulmonary disease (COPD), asthma, anxiety, depression, frequent confusion or agitation, craniofacial morphological alterations, alcohol and drug abuse. Symptoms category included difficulty staying awake during an activity, difficulty concentrating, difficulty in expressing oneself, use of stimulants, interference with work, interference with social relationships, slow reactions and difficulty keeping attention up, difficulty in paying attention to several tasks at once, striving not to make mistakes, and need to doze off.

### The Berlin Questionnaire

The BQ (7) is the most widely used non-invasive screening tool for OSA diagnosis devised to identify subjects with a high likelihood of having OSA based on the frequency, loudness, disturbance and breathing interruptions of nocturnal snoring, on daytime sleepiness, and on the presence of high blood pressure/obesity. The BQ consists of three categories of questions related to the risk of having sleep apneas. Patients can be classified into high-risk or low-risk based on their responses to the individual items and their overall scores in the symptom categories. Category 1 contains five items and incorporates questions about snoring; Category 2 contains three items investigating daytime somnolence; Category 3 contains one item assessing hypertension and information about the Body Mass Index (BMI). Scores from the first two categories were positive if the responses indicated frequent symptoms, such as more than 3–4 times per week, whereas the score from the third category was positive if there was a history of hypertension or a BMI > 30 Kg/m^2^ (7). The overall score was determined from the response to the three categories. Patients were scored as being at OSA high-risk when they had a positive score on two or more categories, else they were considered as being at low-risk (7).

### Statistical Analysis

The answers of all respondents to the BQ were analyzed using descriptive statistics. To identify items associated with being at high-risk of OSA, baseline characteristics, working information, health status, and symptoms category were separately studied in the two OSA risk groups. Continuous variables were summarized by mean and standard deviation (SD) and categorical variables by frequencies and percentages. Kruskal Wallis test and Mann-Whitney U-test were used for assessing difference between high vs. low risk of having OSA. Contingency tables were also analyzed, and chi-square and Fisher's exact tests were carried out to ascertain the presence of relations between the two OSA risk groups. A *p*-value <0.05 was considered statistically significant. BQ scoring and statistical analyses were conducted for all qualitative and quantitative variables using Matlab software.

### Predictive Value

Calculating group statistics is important to establish the statistical relevance of variables in a diagnostic problem so that risk factors or relationships with comorbidities can be assessed. Nonetheless, it is well known (8–10) that relevance is not a synonym for discriminant power, the latter being most useful in classification and prediction: significant variables in a statistical model do not guarantee prediction performance, and non-significant attributes might reveal predictive. For this reason, we decided to also study both Berlin and our questionnaires from the point of view of their prediction capabilities, by techniques related to supervised feature selection and Machine Learning.

It must be noted that prediction in this case is not related to actual OSA diagnosis, because the only data on which we worked is the response to the questionnaires: therefore, the target variable was simply the high risk of being affected by OSA according to the result of the BQ. As the latter is not a perfect test and can give FP and FN (11, 12), our conclusions are valid within the same limits.

XGBoost (13) in python was chosen as the classifier model. A relevant reason was that the responses to the questionnaires unfortunately had a certain number of missing answers and out-of-the-box XGBoost deals quite satisfactorily with missing data thanks to the algorithm called “sparsity-aware split finding”: therefore no explicit imputation mechanism (14) had to be implemented. Moreover, XGBoost is fast and reliable, as also witnessed by frequent wins on Kaggle competitions with this classifier^1^.

After converting the ordered response scales to numeric, the following analysis were performed. First, the Fisher score (15) was calculated on each variable. This index measures the ratio between the inter-class distance and the total intra-class variance, $F={\left({\bar{x}}_{1}-{\bar{x}}_{2}\right)}^{2}/\left(\sigma_{1}^{2}{+\sigma }_{2}^{2}\right)\;$ where ${\bar{x}}_{j}$ and $\sigma_{j}^{2}$ are the mean and the variance of a variable for class *j*. *F* is a parameter clearly related to the discrimination power of each attribute. Similarly, the area under the ROC (Receiver Operating Characteristics) curve (AUROC) for each variable was computed, directly measuring its predictive power. The Fisher score and the AUCROC have similar meaning but they are independent, so they complement each other. However, though these two figures of merit are important because they assess the discriminant power of each feature individually, nonetheless they only partially characterize the dataset, as they neglect the combination of features, which means evaluating two or more features together: it often happens that the scores for single features is low but their combination is strongly discriminant, so some mechanism of feature group scoring assessment is necessary. For this purpose, we employed the backward Sequential Feature Selector (bSSF) from scikit-learn^2^, with XGBoost as the scorer, to build a plot of AUROC vs. the cardinality of the optimal subset of features, from which we could infer interesting conclusions on the prediction power of feature combinations. We finally performed some *ad-hoc* calculations on particular subsets of features, which we considered interesting.

The feature selection procedure based on bSSF was built as follows. We started from the whole dataset of feature vectors containing *n* attributes. The dataset was randomly split into two parts, one for feature selection (*P*1*)* and the other for quality assessment (*P*2) of each subset of selected features. Proportions between selection and quality assessment datasets were arbitrarily set to 70 and 30% of the whole dataset, respectively.

At the *m*-th step (*m* going from 0 to *n* – 2), feature selection by bSSF, from *n* – *m* to *n* – *m –* 1 features, was applied on the *P*1 dataset, followed by prediction quality measurement on the selected features. Therefore, each iteration took as its input the dataset containing the “best” features, as selected by the preceding iteration. At each iteration (with fixed *m*), instead of performing feature selection just once, we preferred to study the robustness of the selected subset of features, by applying bSSF a given number of times (typically 100), each time recording which feature was considered as the least important (downvoted). As the *P*1 vectors were shuffled before bSSF application, we had a certain variability on the selected features and, at the end of this internal loop, we removed the feature that had been downvoted more often.

At this point, with a robust subset of features, we calculated the AUROC (arbitrarily with 50 iterations) on the quality assessment dataset *P*2 and assigned the average AUROC (with an uncertainty calculated as the standard deviation) to the feature set.

The loop on *m* then continued, until there was just one feature in the dataset.

The results of this process were:

- A graph showing AUROC as a function of the number of selected features.
- A list of features, ordered by importance (considering that the least predictive variables, in a multivariate framework, were discarded first).

The whole procedure was repeated many times, each time modifying the initial split between *P*1 and *P*2, so that the influence of random splitting might be judged.

### Ethical Considerations

The ethical aspects of the study were set out in the questionnaire presentation, which was designed in accordance with the principles of the Italian data protection authority (DPA). It was emphasized that participation was voluntary and that the participant could refuse participation in the protocol whenever he or she wished. Those who were interested in participating were given an informed consent form, which recalled the voluntary nature of participation, as well as the confidentiality and anonymous nature of the information.

## Results

### Sample Demographics

Out of 405 people to whom the BQ was administered, the response rate was 95% (*n* = 387). Women were 292 (75% of respondents) and 184 (47.5%) were over 40 years old. The median BMI was 25.4 Kg/m^2^ (range 18–46 Kg/m^2^).

### Berlin Questionnaire Score and Metrics

The BQ was evaluated for all respondents and data were collected (Table 1). According to the questionnaire, the subjects were stratified into low vs. high OSA risk groups by means of a score calculation. Among all subjects, 76 (20%) were categorized as high likelihood of having OSA. Table 2 shows the BQ answer counts subdivided between low and high Berlin score subjects.

**Table 1:** The Berlin questionnaire evaluated for all respondents.

		***N***	**%**
**B1**	**Do you snore?**		
	No	166	43
	Do not know	66	17
	Yes	155	40
**B2**	**If you answered “yes”**		
	Slightly louder than breathing	249	64
	As loud as talking	13	3
	Louder than talking	23	6
	Very loud—it can be heard from adjacent rooms	29	7
	Missing	73	20
**B3**	**How often do you snore?**		
	Never or almost never	174	45
	1–2 times a month	51	13
	1–2 times a week	50	13
	3–4 times a week	33	9
	Every day	79	20
**B4**	**Has your snoring ever bothered other people?**		
	No	181	47
	Do not know	77	20
	Yes	129	33
**B5**	**Has anyone noticed that you stop breathing during your sleep?**		
	Never or almost never	340	88
	1–2 times a month	13	3
	1–2 times a week	14	4
	3–4 times a week	11	3
	Every day	9	2
**B6**	**How often do you feel tired or fatigued after your sleep?**		
	Never or almost never	125	32
	1–2 times a month	82	21
	1–2 times a week	77	20
	3–4 times a week	39	10
	Every day	64	17
**B7**	**During your waking time, do you feel tired, fatigued or not up to par?**		
	Never or almost never	84	22
	1–2 times a month	88	23
	1–2 times a week	99	25
	3–4 times a week	50	13
	Every day	66	17
**B8**	**Have you ever nodded off or fallen asleep while driving a vehicle?**		
	No	342	88
	Do not know	0	0
	Yes	45	12
**B9**	**How often does this occur?**		
	Never or almost never	249	64
	1–2 times a month	22	6
	1–2 times a week	10	2
	3–4 times a week	3	1
	Every day	3	1
	Missing	100	26
**B10**	**Do you have high blood pressure?**		
	No	317	82
	Do not know	15	4
	Yes	55	14

**Table 2:** Berlin questionnaire items between low and high Berlin score (low vs. high OSA risk groups).

		**Low score (*n* = 311) *N* (%)**	**High score (*n* = 76) *N* (%)**	***p*-value**
**B1**	**Do you snore?**			<0.001^***^
	No	164 (53%)	2 (3%)	
	Yes	64 (21%)	72 (95%)	
	Do not know	83 (27%)	2 (3%)	
**B2**	**If you answered “yes”**			<0.001^***^
	Slightly louder than breathing	213 (68%)	35 (46%)	
	As loud as talking	5 (2%)	8 (11%)	
	Louder than talking	11 (4%)	12 (16%)	
	Very loud—it can be heard from adjacent rooms	8 (3%)	21 (28%)	
	missing	74 (24%)	0	
**B3**	**How often do you snore?**			<0.001^***^
	Never or almost never	173 (56%)	1 (1%)	
	1–2 times a month	46 (15%)	5 (7%)	
	1–2 times a week	41 (13%)	9 (12%)	
	3–4 times a week	20 (6%)	13 (17%)	
	Every day	31 (10%)	48 (63%)	
**B4**	**Has your snoring ever bothered other people?**			<0.001^***^
	No	172 (55%)	9 (12%)	
	Yes	68 (22%)	61 (80%)	
	Do not know	71 (23%)	6 (8%)	
**B5**	**Has anyone noticed that you stop breathing during your sleep?**			<0.001^***^
	Never or almost never	299 (96%)	41 (54%)	
	1–2 times a month	3 (1%)	10 (13%)	
	1–2 times a week	3 (1%)	11 (14%)	
	3–4 times a week	3 (1%)	8 (11%)	
	Every day	3 (1%)	6 (8%)	
**B6**	**How often do you feel tired or fatigued after your sleep?**			0.0018^**^
	Never or almost never	117 (38%)	8 (11%)	
	1–2 times a month	73 (23%)	9 (12%)	
	1–2 times a week	68 (22%)	9 (12%)	
	3–4 times a week	23 (7%)	16 (21%)	
	Every day	30 (10%)	34 (45%)	
**B7**	**During your waking time, do you feel tired, fatigued or not up to par?**			0.0029^**^
	Never or almost never	76 (24%)	8 (11%)	
	1–2 times a month	82 (26%)	6 (8%)	
	1–2 times a week	88 (28%)	11 (14%)	
	3–4 times a week	33 (11%)	17 (22%)	
	Every day	32 (10%)	34 (45%)	
**B8**	**Have you ever nodded off or fallen asleep while driving a vehicle?**			<0.001^***^
	No	284 (91%)	58 (76%)	
	Yes	27 (9%)	18 (24%)	
**B9**	**How often does this occur?**			<0.001^***^
	Never or almost never	212 (68%)	37 (49%)	
	1–2 times a month	15 (5%)	7 (9%)	
	1–2 times a week	5 (2%)	5 (7%)	
	3–4 times a week	1 (0%)	2 (3%)	
	Every day	0	3 (4%)	
	missing	78 (25%)	22 (29%)	
**B10**	**Do you have high blood pressure?**			<0.001^***^
	No	284 (91%)	33 (43%)	
	Yes	16 (5%)	39 (51%)	
	Do not know	11 (4%)	4 (5%)	

** *p < 0.01*;

*** *p < 0.001*.

*A p-value < 0.05 was considered statistically significant*.

Respondents were also asked if they had already been diagnosed for OSA through a gold standard test (e.g., polysomnography). Among the subjects identified as high-risk, 24% (*n* = 18, 5% of the complete sample) had already been diagnosed with OSA whereas 76% (*n* = 58, 15% of the sample) had not undergone any diagnostic test. Among the subjects categorized as low-risk for OSA, 1% (*n* = 2), had received a diagnosis of OSA (false negatives) whereas 99% had not been tested.

As reported in the literature (16), the dominant symptom of OSA is snoring with a prevalence of 75–90%. Accordingly, in our sample the high-risk OSA group had a significantly larger proportion of respondents reporting frequent snoring (95%) compared to the low-risk group (21%). Nocturnal snoring also increased in frequency and loudness in high-risk OSA cases compared with low-risk, and this difference was statistically significant (*p* < 0.001 for both). Specifically, 28% of the high-risk group report snoring very loudly compared with 3% of the low-risk group. The percentage of those who snore every night also increases from 10 to 63% in the high-risk group.

Nocturnal symptoms may also include apnea and dyspnea generally observed by bed partners and this was confirmed by the bothersome snoring percentage that passed from 22% in the low-risk to 80% in the high-risk group. These differences were statistically significant (*p* < 0.001).

The high-risk group also reported more breathing interruptions than the low-risk subjects (*p* < 0.001).

Fatigue, somnolence at awakening and during daytime are also symptoms significantly present in the high-risk group compared to the low risk group (*p* = 0.0018 and 0.0029, respectively). The percentage of those who reported falling asleep while driving a vehicle was also higher in the high-risk group (24%) than for the low-risk subjects (9%), with a statistically significant difference (*p* < 0.001).

This significance is also present in the frequency of episodes (*p* < 0.001).

High blood pressure was also reported in half of the high risk subjects (51%) compared with 5% of the low risk ones, and this difference was statistically significant.

Socio-demographic characteristics, work information, clinical factors, and symptoms category were compared between the two OSA risk groups. The results are summarized in Table 3.

**Table 3:** Baseline characteristics of nurses between low and high Berlin scores (low vs. high OSA risk groups).

	**Items**	**Low score** **(*n* = 311)**	**High score** **(*n* = 76)**	***p*-value**
		***N* (%)**	***N* (%)**	
**Anamnesis factors**				
	**Q1 Gender**			<0.001^***^
	Female	247 (79%)	46 (61%)	
	Male	64 (21%)	30 (39%)	
	**Q2 Age (Y)**			0.0011^**^
	21–30	103 (33%)	12 (16%)	
	31–40	75 (24%)	13 (17%)	
	41–50	68 (22%)	32 (42%)	
	51–60	62 (20%)	16 (21%)	
	>61	3 (1%)	3 (4%)	
	**Q3 BMI group (Kg/m** ^**2**^ **)**			<0.001^***^
	Underweight <18.5	6 (2%)	2 (3%)	
	Normal weight 18.5–24.9	179 (58%)	25 (33%)	
	Overweight 25–29.9	73 (23%)	27 (36%)	
	Obese ≥ 30	53 (17%)	22 (29%)	
	**Q4 Smoking**			0.946
	Yes	81 (26%)	23 (30%)	
	No	190 (61%)	39 (51%)	
	Ex-smoker	40 (135)	14 (18%)	
	**Q5 Neck circumference (cm)**			0.834
	<43 men/41 women	135 (43%)	29 (38%)	
	≥43 men/41 women	18 (6%)	11 (14%)	
	Unknown	158 (51%)	36 (47%)	
**Working information**				
	**Q6 Profession**			0.039^*^
	Nurse	282 (91%)	63 (83%)	
	Coordinator	22 (7%)	5 (7%)	
	Executive	1 (0%)	6 (8%)	
	Other	6 (2%)	2 (3%)	
	**Q7 Instruction level**			0.049^*^
	Regional Diploma	61 (20%)	24 (32%)	
	University Diploma	20 (6%)	7 (9%)	
	Bachelor's degree	167 (54%)	31 (41%)	
	Master degree	34 (11%)	8 (11%)	
	Post-graduate	29 (9%)	6 (8%)	
	**Q8 Work experience (Y):**			0.0072^**^
	1–5	118 (38%)	12 (16%)	
	6–10	32 (10%)	15 (20%)	
	11-15	29 (9%)	2 (0%)	
	16–20	30 (8%)	13 (17%)	
	21–25	27 (12%)	15 (20%)	
	26–30	37 (10%)	8 (10%)	
	>31	38 (12%)	11 (14%)	
	**Q9 Working hours**			0.325
	Full-time	284 (91%)	72 (95%)	
	Part-time	27 (9%)	4 (5%)	
	**Q10 Work shift**			0.758
	Daily shift only	129 (41%)	33 (43%)	
	24 h shift	182 (57%)	43 (57%)	
	**Q11 Work shift regularity**			0.277
	Yes	193 (62%)	42 (55%)	
	No	118 (38%)	34 (45%)	
**Clinical factors**				
	**Q12 Previous OSA diagnosis**			<0.001^***^
	Yes	2 (1%)	18 (24%)	
	No	309 (99%)	58 (76%)	
	**Q13 Hypo/hyperthyroidism**			<0.001^***^
	No	265 (85%)	60 (79%)	
	Yes	46 (15%)	16 (21%)	
	**Q14 Arrhythmias**			<0.001^***^
	No	272 (87%)	48 (63%)	
	Yes	39 (13%)	28 (37%)	
	**Q15 Transient ischemic attack or stroke**			0.0013^**^
	No	310 (100%)	71 (93%)	
	Yes	1 (0%)	5 (7%)	
	**Q16 Diabetes mellitus**			<0.001^***^
	No	305 (98%)	65 (86%)	
	Yes	6 (2%)	11 (14%)	
	**Q17 Presence of cerebrovascular diseases**			0.022^*^
	No	309 (99%)	73 (96%)	
	Yes	2 (1%)	3 (4%)	
	**Q18 Anxiety**			0.0014^**^
	No	207 (66%)	35 (46%)	
	Yes	104 (33%)	41 (54%)	
	**Q19 Sleep disorders**			<0.001^***^
	No	239 (77%)	38 (50%)	
	Yes	72 (23%)	36 (50%)	
	**Q20 Chronic obstructive pulmonary disease (COPD)**			<0.001^***^
	No	310 (100%)	70 (92%)	
	Yes	1 (0%)	6 (8%)	
	**Q21 Asthma**			0.0018^**^
	No	291 (94%)	58 (76%)	
	Yes	30 (6%)	18 (24%)	
	**Q22 Frequent confusion or agitation**			0.022^*^
	No	303 (97%)	68 (89%)	
	Yes	8 (3%)	8 (11%)	
	**Q23 Alcohol abuse**			0.022^**^
	No	308 (99%)	71 (93%)	
	Yes	3 (1%)	5 (7%)	
	**Q24 Drug abuse**			0.0061^*^
	No	307 (99%)	71 (93%)	
	Yes	4 (1%)	5 (7%)	
	**Q25 Depression**			0.0082^*^
	No	280 (90%)	60 (79%)	
	Yes	31 (10%)	16 (21%)	
	**Q26 Craniofacial morphological alterations**			0.66
	No	305 (98%)	74 (97%)	
	Yes	6 (17)	2 (3%)	
**Symptoms category**				
	**Q27 Have you ever fallen asleep during an activity (e.g., during work)?**			<0.001^***^
	Never	284 (91%)	58 (76%)	
	About once a week	20 (6%)	8 (11%)	
	Two or three times a week	3 (1%)	5 (7%)	
	Almost every day	3 (1%)	3 (4%)	
	Several times a day	1 (0%)	2 (3%)	
	**Q28 Did you have difficulty concentrating during an assignment?**			<0.001^***^
	Never	152 (49%)	22 (29%)	
	About once a week	117 (38%)	22 (29%)	
	Two or three times a week	33 (11%)	14 (18%)	
	Almost every day	7 (2%)	11 (14%)	
	Several times a day	2 (1%)	7 (9%)	
	**Q29 Did you have to force yourself to express yourself clearly?**			0.012^**^
	Never	195 (63%)	36 (47%)	
	About once a week	89 (29%)	16 (21%)	
	Two or three times a week	16 (5%)	15 (20%)	
	Almost every day	8 (3%)	6 (8%)	
	Several times a day	3 (1%)	3 (3%)	
	**Q30 Have you had to use stimulants (coffee, tea, ginseng, etc.) to stay active?**			0.0052^**^
	Never	132 (42%)	21 (28%)	
	About once a week	62 (20%)	12 (16%)	
	Two or three times a week	29 (9%)	13 (17%)	
	Almost every day	69 (22%)	−28%	
	Several times a day	19 (6%)	9 (12%)	
	**Q31 Have the problems reported in the previous questions interfered with your ability to work?**			<0.001^***^
	I have not had these problems	138 (44%)	30 (39%)	
	Never	112 (36%)	15 (20%)	
	About once a week	48 (15%)	15 (20%)	
	Two or three times a week	8 (3%)	12 (16%)	
	Almost every day	4 (1%)	3 (4%)	
	Several times a day	1 (0%)	1 (1%)	
	**Q32 Have the problems reported in the previous questions interfered with your social relationships?**			<0.001^***^
	I have not had these problems	133 (43%)	15 (20%)	
	Never	104 (33%)	27 (36%)	
	About once a week	58 (19%)	17 (22%)	
	Two or three times a week	9 (3%)	11 (14%)	
	Almost every day	6 (2%)	5 (7%)	
	Several times a day	1 (0%)	1 (1%)	
	**Q33 Have your reactions in everyday situations been slow?**			<0.001^***^
	Never	189 (61%)	34 (45%)	
	About once a week	92 (30%)	17 (22%)	
	Two or three times a week	22 (7%)	15 (20%)	
	Almost every day	4 (1%)	7 (9%)	
	Several times a day	4 (1%)	3 (4%)	
	**Q34 Did you have to try harder than usual to keep track of what you were doing?**			<0.001^***^
	Never	169 (54%)	26 (34%)	
	About once a week	106 (34%)	23 (30%)	
	Two or three times a week	19 (6%)	14 (18%)	
	Almost every day	14 (5%)	7 (9%)	
	Several times a day	3 (1%)	6 (8%)	
	**Q35 Did you have difficulty paying attention for a long time on a task?**			<0.001^***^
	Never	180 (58%)	26 (34%)	
	About once a week	105 (34%)	25 (33%)	
	Two or three times a week	17 (5%)	13 (17%)	
	Almost every day	6 (2%)	10 (13%)	
	Several times a day	17 (5%)	2 (3%)	
	**Q36 Have you had difficulty paying attention to multiple tasks at once (e.g., listening to a radio program while**			0.0033^**^
	**driving a car)?**			
	Never	208 (67%)	41 (54%)	
	About once a week	82 (26%)	15 (20%)	
	Two or three times a week	9 (3%)	10 (13%)	
	Almost every day	12 (4%)	8 (11%)	
	Several times a day	0	2 (3%)	
	**Q37 Did you have to work hard to pay attention and not make mistakes?**			<0.001^***^
	Never	165 (53%)	26 (34%)	
	About once a week	115 (37%)	25 (33%)	
	Two or three times a week	16 (5%)	12 (16%)	
	Almost every day	11 (4%)	11 (14%)	
	Several times a day	4 (1%)	2 (3%)	
	**Q38 Did you feel the need to doze off during the course of the day?**			<0.001^***^
	Never	79 (25%)	5 (7%)	
	About once a week	99 (32%)	23 (30%)	
	Two or three times a week	69 (22%)	18 (24%)	
	Almost every day	61 (20%)	27 (36%)	
	Several times a day	3 (%)	3 (4%)	

* *p < 0.05*;

** *p < 0.01*;

*** *p < 0.001*.

*Anamnesis factors, work information, clinical status, and symptoms category were assessed. A p-value <0.05 was considered statistically significant*.

### Predictive Value of the Berlin Questionnaire Variables

#### Fisher Indices and AUROC for Single Variables

The ten variables from the BQ plus BMI were considered. The most discriminant variables were the four related to snoring (B1 to B4 in Table 1) with B1 being the most important in absolute (AUROC = 0.88, *F* = 1.9) and snoring loudness B2 being the least predictive. As to the two variables with relatively objective measurement, i.e., having high blood pressure, B10, and the body mass index (computed from the subject physical data), the former had high predictivity (AUROC = 0.74, *F* = 0.80) while the latter showed lower discriminant power (AUROC = 0.64, *F* = 0.03). This result was quite surprising if compared with the one reported in (18) where BMI is found to be quite a strong predictor.

#### Sequential Feature Selection

**Figure 1:**
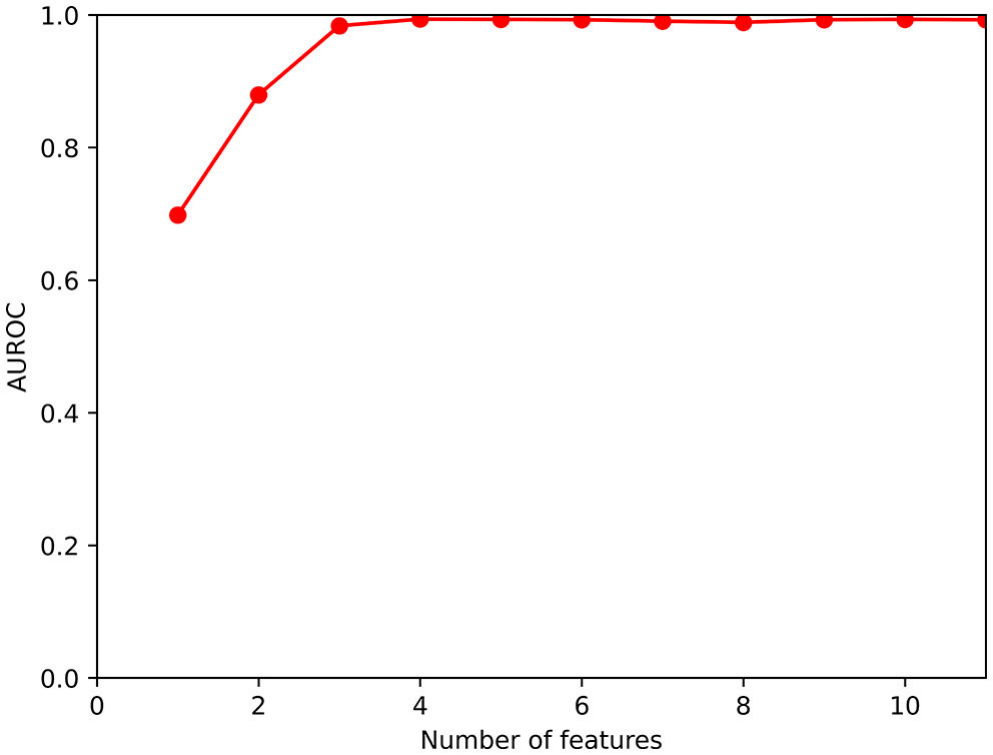
Functional dependence of AUROC on the cardinality of the set of selected features.

The typical relationship between the number of features and AUROC we obtained by the bSSF procedure is shown in Figure 1. Repeating the run with different random splits of *P*1 vs. *P*2 partitioning did not appreciably change the result, with AUROC for sets ≥ 3 features always attaining values near 1. Reaching so high AUROC with the full set of variables, of course, has no particular meaning because the target variable (high risk of OSA) is obtained from the BQ variables (the answers to the questions), so there exists a well-established *a priori* relationship between the variables and the target, which the classifier finds. On the other hand, what is surprising is the fact that a subset of three variables is capable of predictive power comparable to the whole questionnaire.

The subset of three variables was reasonably robust and did not depend too much on the particular dataset split; after about 60 runs, the subset was found to contain the variables computed from B10 (selected at every run), B1 (present in 73% of the “best” feature subsets), B6 (presence in 38%), B7 (37%), B3 (25%), B4 (2%). We remark that hypertension B10 is always among the most useful features [which was already known from the single-variable calculations; this result confirms what was found in (18)]. Considering now the remaining five features, three concern snoring (B1, the most voted after B10; then B3 and B4) while two concern feeling tired in daytime, either at wake-up or along the day (B6 and B7), with similar presence in the subsets. By calculating the (normalized) co-occurrence matrix of these five variables in the “best” feature subsets:


$$COM=\begin{array}{c} B1\\ B6\\ B7\\ B3\\ B4 \end{array}\left(\begin{array}{ccccc} 0.183 & 0.100 & 0.083 & 0 & 0\\ 0.100 & 0.158 & 0 & 0.054 & 0.004\\ 0.083 & 0 & 0.092 & 0.008 & 0\\ 0 & 0.054 & 0.008 & 0.062 & 0\\ 0 & 0.004 & 0 & 0 & 0.004 \end{array}\right)$$

It is evident that B1 is always accompanied by B6 or B7, so two natural choices for three-variate subsets of features could be {B1, B6, B10}, immediately followed by {B1, B7, B10}. After selecting these “best” sets of variables, good practice would require verifying the conclusions on an independent test dataset. Being this impossible at this time for lack of data, their predictive values was recalculated on the whole available dataset, with 5-fold cross validation. AUROC were 0.98 for both.

### Predictive Value for the Proprietary Questionnaire Variables

The proprietary questionnaire was also examined from the Machine Learning point of view, with a similar approach but very different results. The target variable was, as in the preceding analysis, the BQ output in terms of high vs. low risk of OSA. Global AUROC was not too high, with values about 0.80, which witnesses the relationship between the questions and the pathology, but also the scarce usefulness of the proprietary questionnaire in a ML context, at least with the data we possess. No variable derived from the questionnaire items revealed to be strikingly discriminant *per se*. Moreover, the partially stochastic nature of the feature selection process (due to the different random choices of the selection and quality assessment sets, respectively, *P*1 and *P*2), leaded to quite different AUROC vs. number of features functional dependences at each run (in which AUROC slowly decreased from 80 to 60% with the progressive depletion of the feature set).

## Discussion

Of 387 screened patients who completed the BQ, about 20% (*n* = 76) fell within the high-risk group. Socio-demographic characteristics, work information, clinical factors, and symptoms category were compared between the two groups and are reported in Table 2.

### Socio-Demographic Baseline Characteristics

Age is a well-established risk factor for OSA (19, 20). The increase in the prevalence of OSA with age could be explained in part by the increase in comorbidities, menopause, hypertension, BMI, but also by the decrease of tongue and palate muscle functions and activities that occurs in older adults (21, 22). Regarding the age of the sample, in the high-risk group 67% (*n* = 51) was ≥41 years old compared to 41% (*n* = 133) in the low-risk group. We have to consider that our cohort is predominantly composed of young subjects, more than half being <40 years old and only <2% of subjects being more than 60 years old. In our cohort, age was also found to be a risk factor significantly associated with high risk of OSA (*p* < 0.0001).

With respect to gender, epidemiological studies reported a prevalence ranging from 13 to 31% in men and 4 to 21% in women (17, 23–27). It is difficult to confirm this prevalence in our analysis, considering that our sample is predominantly female (76%). Despite this, we found a statistically significant difference between low-risk and high-risk groups with respect to gender (*p* = 0.0011). In particular, the percentage of men increases from 21% at low-risk to 39% at high-risk. In contrast, the percentage of women at low-risk is 79% and decreases in high-risk subjects (61%).

Obesity is the most severe known risk factor for OSA. Generally, almost 60% of patients with OSA are obese (28). The risk of OSA increases progressively with BMI and also with neck circumferences (29). In our analysis, the mean of BMI was significantly higher in the high-risk group than in the low-risk group (*p* < 0.001). Regarding neck circumferences, half of subjects did not know their neck circumferences. However, neck circumferences were higher than the chosen cut-off in the high-risk group (14%) compared to the low-risk group (6%).

No association was found with smoking and OSA in our sample and this reflects what is found in the literature (30). However, inhalation of cigarette smoke increases oxidative stress and systemic inflammation, which are typically present in OSA (30). Thus, the concomitant presence of OSA in smoker could worsen disease progression.

### Work Information

Regarding work information, only the number of years of work experience seems to be associated with a high risk of OSA. However, rather than being a risk factor *per se*, this variable could be significant just because it is correlated with increasing age, an important risk factor previously discussed. Distribution of working time (full time/part time), work shift (day shift only or 24 h shift) and work shift regularity (yes/no) were not found to be associated with a high risk of OSA. Interestingly, professional categories and instruction level appear to be determinants between the two groups (0.039 and 0.049, respectively).

### Health Status

Among all the clinical factors investigated, only the presence of craniofacial morphological alterations was not found to be a risk factor associated with an elevated risk of OSA, contrary to what reported in the literature (31). However, we must consider that only 8 subjects declared to have these alterations, which makes the sample less significant. Sleep disorders, instead, were obviously statistically significant between the two groups (*p* < 0.001), demonstrating the reliability of the sample.

Hypertension was already known to be associated with OSA (32, 33). Normally, 50% of hypertensive patients have OSA and this percentage rises to 85% in patients with hypertension who have at least another OSA symptom (34, 35). Subjects with OSA have an 1.8-times increased risk of resistant hypertension compared to non-OSA individuals (36). Our sample confirmed these data since 51% of high-risk persons were hypertensive compared with 5% found in low-risk subjects.

Arrhythmias and transient ischemic attack or stroke were found to be associated to high OSA risk score (*p* < 0.001 and *p* = 0.0013, respectively). This is in line with the literature, which attests that prevalence of OSA is estimated to be between two and three times higher in patients with cardiovascular diseases (37).

The percentage of OSA patients who suffer from type 2 diabetes was about 30% (*n* = 118). The link between diabetes and OSA seems bidirectional but has not been fully evaluated yet. In our cohort, 14% of the high-risk group shows presence of diabetes mellitus, compared to 2% of patients found in the low-risk group. This is statistically significant and the association between diabetes mellitus and being at high-risk is also significant (*p* < 0.001).

OSA and asthma are closely related. Numerous studies have consistently reported higher OSA burden among subjects with asthma (38, 39) and in relation to asthma severity (38, 40). In our sample, the percentage of individuals with asthma in the low-risk group was 6% rising to 24% in high-risk group. Asthma was also found to be a strong risk factor for OSA (*p* = 0.0018).

Chronic obstructive pulmonary disease (COPD) is also highly associated with OSA. COPD is one of the most prevalent respiratory diseases worldwide. There exists what is called COPD-OSA overlap syndrome that represents a distinct clinical diagnosis, where clinical outcomes are even worse than in each disease alone (41). Based on this evidence, we found a significant difference between the low and high-risk groups (*p* < 0.001).

Recent systematic reviews and meta-analyses reported that OSA is linked to depression (42) and anxiety (43). Other longitudinal studies suggested that patients with OSA are about twice as likely to be depressed than those without OSA (44, 45). In our sample, the rate of depression increased from 10% in the low-risk group to 21% in the high-risk OSA group, while the rate of anxiety increased from 33 to 54%. We also found a strong correlation between being at high-risk of OSA and having both depression and anxiety (*p* = 0.0014 and *p* = 0.0082, respectively).

Frequent confusion and agitation resulted also to be an important risk factor (*p* = 0.0022) in our cohort. In particular, 11% of the high-risk subjects show presence of confusion and agitation, compared to 3% of those found in the low-risk group. This phenomenon could be related to anxious behavior, but several efforts should be done for understanding this association.

Excessive alcohol consumption and drug abuse were also assessed between low vs. high score. Results from the literature revealed that alcohol consumption is associated with 25% increased risk of OSA (46). To the best of our knowledge, no data was shown for drug abuse. We found that 7% of the high-risk group declared alcohol and drug abuse, compared to 1% of patients found in the low-risk group. Alcohol and drug abuse were also found to be two independent risk factors for the high-risk group (*p* = 0.0022 and *p* = 0.0061, respectively).

### Symptoms Category

Daytime OSA symptoms consist of unexplained fatigue and excessive sleepiness. Patients also report repetitive problems with concentration and memory as well as depressive symptoms (47) and impairment of cognitive functions (48). Moreover, a study of men and women aged 60 years and older showed memory impairment related to OSA and hypertension (49). All of these evidences are in line with our findings: difficulty staying awake during an activity, difficulty concentrating, difficulty in expressing oneself, use of stimulants, interference with work, interference with social relationships, slow reactions and difficulty keeping attention up, difficulty in paying attention to several tasks at once, striving not to make mistakes, and need to doze off, are all significantly strong risk factors related to high-risk of having OSA. These symptoms fully describe the OSA patient during his/her daily activity, including working and social activities.

### Predictive Value of Questionnaire Items

As concerns the predictive value of the variables acquired by the BQ, our conclusion was that a reduced set of questions, i.e., a reduced set of selected features, composed only of Table 4, is sufficient to obtain an output close to that of the BQ, by using a trained XGBoost classifier.

**Table 4:** Simplified Berlin questionnaire.

**B1**	**“Do you snore?”**	**No/do not know/yes**
B6	“How often do you	Never or almost never
	feel tired or fatigued	1–2 times a month
	after your sleep?”	1–2 times a week
		3–4 times a week
		Every day
or		
B7	“During your waking time,	Never or almost never
	do you feel tired,	1–2 times a month
	fatigued or not up to	1–2 times a week
	par?”	3–4 times a week
		Every day
B10	“Do you have high	No
	blood pressure?”	Do not know
		Yes

This reduced questionnaire shows some similarity with the one proposed in Arunsurat et al. (18) with the important difference that (as already remarked) BMI is not preserved in the reduced set. The discrepancy might partly come from the different group considered, i.e., the high percentage of young and prevalently female respondents in our sample compared to the all-male healthcare workers investigated in Arunsurat et al. (18).

From the Results section, it is also evident that the proprietary questionnaire is interesting from the point of view of risk factor assessment, but the ML approach gave no hint on the possibility of replacing/integrating the original Berlin test with (parts of) it. In order to clarify this possibility, a dataset with ground truth coming from PSG or HST is needed along with the questionnaire itself.

### Limits

The results of our study must be considered taking into account some limitations that concern the sample size, the lack of the actual disease diagnosis for most subjects, the absence of disease follow-up and long-term effect investigation for the subjects who declared to suffer from OSA and, finally, the possible reluctance of the respondents to faithfully declare their health status since they are professional nurses. Moreover, our survey group does not fully represent the general population, because of the high percentage of young and prevalently female respondents. Finally, we are also aware that the study might give different conclusions in different ethnic groups, depending on language, habits, lifestyles or physical conformation.

### Conclusions

In conclusion, there are numerous risk factors associated with a high-risk of having OSA in a population of nurses. Given the high percentage of people who are still underdiagnosed for OSA and the lack of knowledge about this disease, our study contributes to highlight an alarming result that may be just the tip of the iceberg. This study could be helpful to expand awareness about it, especially among professional nurses, who are one of the most important categories in health and our care. It could also allow more professionals to investigate suspected patients who could undergo overnight polysomnography, as well as to explore possible alternative screening tests and cures for the treatment of this still too hidden disease.

Further efforts should be done to increase the number of diagnoses but also, more importantly, to refer these subjects for screening. On this regard, our simplified test might also allow a better administration of the questionnaire facilitating the orientation of the subject at risk toward the diagnostic pathway. We plan indeed a prospective clinical trial that can use the simplified Berlin test together with our proprietary questions on the general population, with the aim of possibly creating a richer questionnaire with better sensitivity and specificity.

## Data Availability Statement

The original contributions presented in the study are included in the article, further inquiries can be directed to the corresponding author.

## Ethics Statement

Ethical review and approval was not required for the study on human participants in accordance with the local legislation and institutional requirements. The participants provided their written informed consent to participate in this study.

## Author Contributions

GDN, LC, RL, and LDB contributed to conception and design of the study. LC, GDN, AC, ME, and MC organized the database. LC, GDN, and EV performed the statistical analysis. LC and GDN wrote the first draft of the manuscript. LC, GDN, MA, DT, and LDB wrote sections of the manuscript. All authors contributed to manuscript revision, read, and approved the submitted version.

## Conflict of Interest

The authors declare that the research was conducted in the absence of any commercial or financial relationships that could be construed as a potential conflict of interest.

## Publisher's Note

All claims expressed in this article are solely those of the authors and do not necessarily represent those of their affiliated organizations, or those of the publisher, the editors and the reviewers. Any product that may be evaluated in this article, or claim that may be made by its manufacturer, is not guaranteed or endorsed by the publisher.

## Footnotes

^1^ https://github.com/dmlc/xgboost/tree/master/demo#machine-learning-challenge-winning-solutions

^2^ https://scikit-learn.org/
